# Efficacy and Safety of Co‐Ablation Combined With Aintilimab for Metastatic Melanoma to the Liver (CryoCheck‐001): A Phase II Trial

**DOI:** 10.1002/mco2.70997

**Published:** 2026-09-15

**Authors:** Lujun Shen, Letao Lin, Shuanggang Chen, Chen Li, Ruizhi Tang, Lin Xie, Yujia Wang, Yunpeng Li, Hongliang Zou, Dandan Li, Xiaoshi Zhang, Weijun Fan

**Affiliations:** ^1^ Department of Minimally Invasive Interventional Therapy Sun Yat‐sen University Cancer Center Guangzhou China; ^2^ State Key Laboratory of Oncology in South China Collaborative Innovation Center of Cancer Medicine Sun Yat‐sen University Guangzhou China; ^3^ Department of Interventional Oncology The First Affiliated Hospital of Sun Yat‐Sen University Guangzhou China; ^4^ Department of Biological Therapy Center Sun Yat‐sen University Cancer Center Guangzhou China

**Keywords:** liver metastasis, melanoma, co‐ablation, sintilimab, phase II trial

## Abstract

Liver metastasis is a key driver of immune tolerance in melanoma, limiting immune checkpoint inhibitor efficacy. This prospective single‐arm phase II trial investigated the efficacy and safety of co‐ablation combined with sintilimab for liver‐metastatic melanoma (ChiCTR2200061591). Eligible patients received sintilimab (anti‐PD‐1, 200 mg every 3 weeks) for up to eight cycles alongside co‐ablation. The primary endpoint was a 6‐month progression‐free survival (PFS) rate per Response Evaluation Criteria in Solid Tumors v1.1. Secondary endpoints included overall survival (OS), PFS time, and overall response rate (ORR). Between June 2022 and July 2025, 16 patients were enrolled. Three patients achieved a partial response (ORR 18.75%). The 6‐month PFS rate was 31.25% (95% confidence interval [CI]: 15.1–64.6), with a median PFS of 3.94 months (95% CI: 2.77–14.30). The 12‐ and 24‐month OS rates were 53.8% (95% CI: 33.7–86.0) and 28.7% (10.3–80.1), respectively; median OS was 16.67 months (95% CI: 7.50–NR). Grade ≥ 3 treatment‐related adverse events occurred in 14 of 16 patients (87.5%), the most common being elevated liver enzyme levels. Co‐ablation combined with sintilimab demonstrates a manageable safety profile and encouraging antitumor activity in patients with liver‐metastatic melanoma, meriting further evaluation.

## Graphical abstract

Liver metastasis is a key driver of immune tolerance in patients with melanoma receiving immune checkpoint inhibitor therapy. This single‐arm phase II trial prospectively investigated the efficacy and safety of co‐ablation combined with sintilimab for liver metastatic melanoma. Between June 15, 2022 and July 15, 2025, 16 patients with liver metastatic melanoma were enrolled. A total of three patients had a partial response, with an ORR of 18.75%. The 6‐month PFS rate was 31.25% (95% CI: 15.1%–64.6%), with a median PFS time of 3.94 months (95% CI: 2.77–14.30). The proportion of CD3+CD8+ T cells increased significantly (*p* = 0.042), whereas the proportion of CD19+ B cells decreased (*p* = 0.046) 3 weeks after the combination therapy. All treatment‐related adverse events were managed with symptomatic treatment. Co‐ablation combined with sintilimab demonstrates a manageable safety profile and encouraging antitumor activity in patients with liver metastatic melanoma.

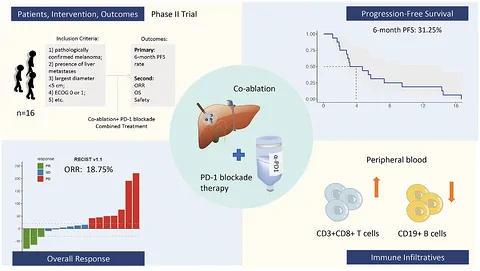

## 1 Background

Over the past decade, programmed cell death receptor 1 (PD‐1) blockade therapy has revolutionized the treatment of advanced‐stage melanoma [1, 2]. However, liver metastases develop in 20%–50% of these patients and have been identified as a key driver of systemic immune tolerance, posing a significant challenge to the efficacy of immunotherapy [3]. The estimated response rate among patients with hepatic metastases from melanoma receiving PD‐1 blockade monotherapy ranges from 0% to 10% [4, 5].

The 2025 National Comprehensive Cancer Network (NCCN) guidelines highlight the increasingly important role of local therapy, including tumor ablation, radiotherapy, and surgical resection, in managing metastatic melanoma. Our earlier ambispective cohort study demonstrated that combining pembrolizumab with cryoablation produced an overall response rate (ORR) of 26.7% in non‐ablated lesions among patients with liver metastasis from melanoma [6]. Our recent Cryo‐Fire study comparing between cryoablation combined with PD‐1 blockade therapy and PD‐1 blockade therapy alone in a large multi‐center cohort confirmed the synergistic effect of cryoablation in augmenting anti‐tumor immune response. The potential mechanisms involved enhanced T‐cell anti‐tumor immunity, natural killer (NK)‐cell‐based innate immunity, and reduced immunosuppression effect caused by a decline in tumor‐associated macrophages [7].

Although cryoablation can enhance anti‐tumor immunity, the high risk of abdominal bleeding when treating liver metastasis limits its widespread adoption [8, 9]. The co‐ablation system, approved by CFDA in 2017, is a novel tumor ablation device that integrates ultra‐low temperature cryoablation and high‐intensity thermal ablation to maximize tumor cell destruction [10]. In the freezing mode, the temperature can be lowered to −196°C, leading to the formation of ice crystals inside and outside cells that cause mechanical damage to cell membranes and organelles [11]. In the heating mode, the temperature can be increased to +80°C, effectively preventing both abdominal bleeding and tumor implantation [12]. Beyond physical ablation, co‐ablation exerts potent immunomodulatory effects by inducing immunogenic cell death, releasing tumor antigens, and reshaping the microenvironment to increase CD8^+^ T‐cell infiltration and convert “cold” tumors into “hot” ones. In the CASTLE‐01 trial, co‐ablation with sintilimab and lenvatinib achieved an ORR of 75% and a median progression‐free survival (PFS) of 16.8 months in advanced cholangiocarcinoma. Furthermore, mechanistic analyses confirmed enhanced tumor immunogenicity, dendritic cell activation, and sustained intratumoral CD8^+^PD1^high^ T‐cell replenishment [13]. Additionally, the BOOSTER trial further supported the cryoablation component's superiority via type I interferon responses [14]. These advantages make the co‐ablation system a superior alternative to conventional argon–helium cryoablation for liver‐metastatic melanoma.

Sintilimab is an anti‐PD‐1 antibody developed in China, notable for its distinct binding affinity and epitope [15]. Sintilimab targets the FG loop of PD‐1, exhibiting a binding affinity approximately 50 times stronger than pembrolizumab. As a fully humanized monoclonal antibody, sintilimab also has lower immunogenicity, potentially minimizing immune‐related adverse events (irAEs). Importantly, its binding epitope on PD‐1 partially differs from that of nivolumab and pembrolizumab, potentially offering superior signaling blockade efficiency, which may be particularly advantageous for reinvigorating exhausted T cells within the highly suppressive liver metastatic microenvironment [16]. This organ‐specific effect is supported by clinical evidence. Sintilimab, in combination with bevacizumab, is approved for unresectable hepatocellular carcinoma (HCC), after demonstrating clinical efficacy within the hepatic tumor microenvironment [17]. Mechanistically, single‐cell analyses in responding patients with HCC have revealed that sintilimab‐based therapy drives a widespread convergence on interferon signaling across immune lineages, including CD8^+^ T‐cell clonal expansion and M1‐like macrophage polarization, suggesting a capacity to actively remodel the immunosuppressive liver milieu [18].

Therefore, we conducted this phase II trial (CryoCheck‐001) aiming to evaluate the safety and efficacy of co‐ablation combined with sintilimab in patients with liver‐metastatic melanoma. Despite its early termination, this study provided critical clinical evidence that the combined treatment exhibited manageable safety and promising antitumor activity in this high‐risk population.

## 2 Results

### 2.1 Patient Characteristics

**FIGURE 1:**
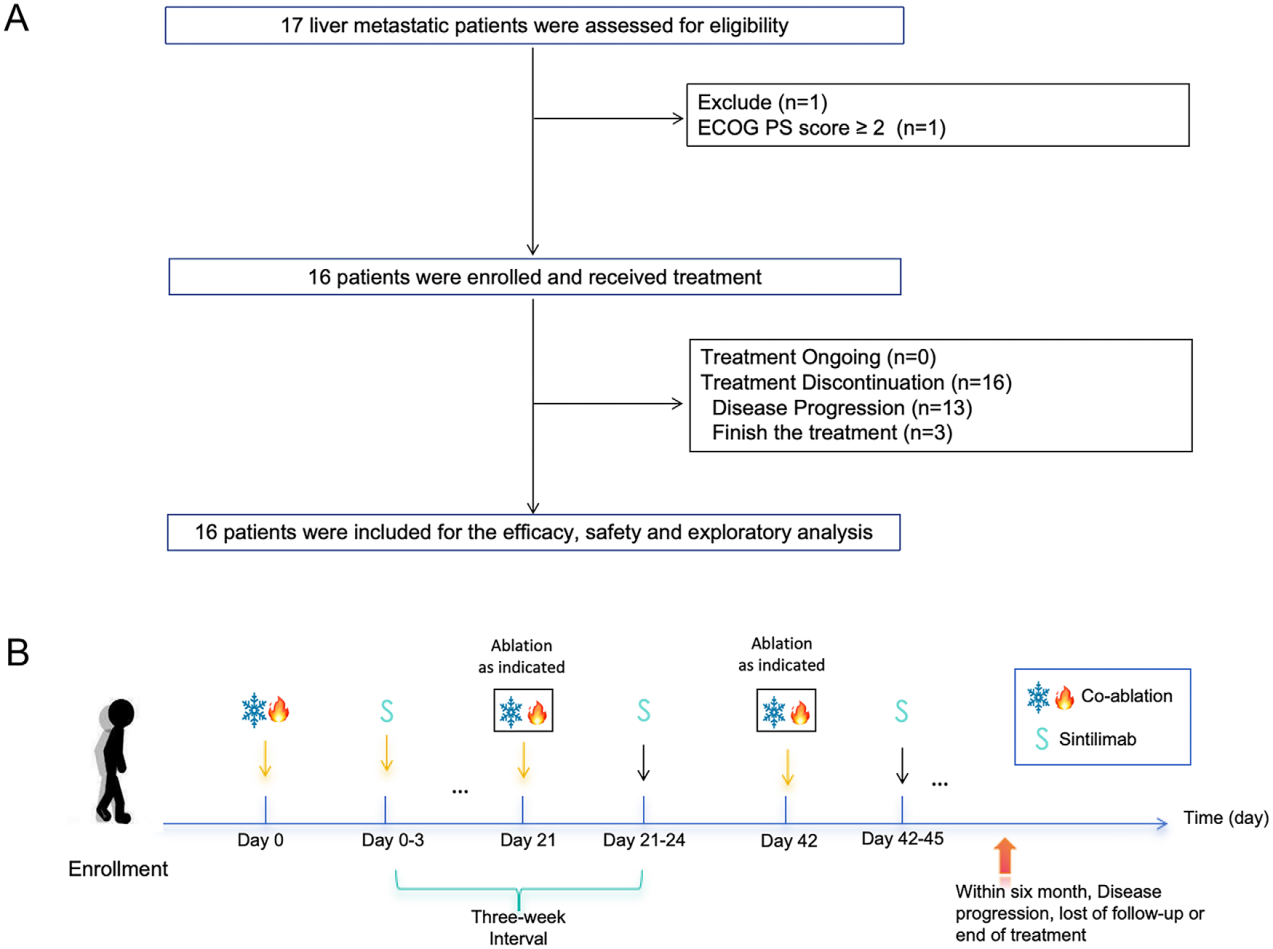
Patient flowchart and treatment schedule. (A) A total of 17 patients with liver‐metastatic melanoma are screened and 16 enrolled. (B) Patients are scheduled to receive co‐ablation and administration of sintilimab every 3 weeks, with a maximum of eight cycles within the time‐frame of 6 months since enrollment.

Between June 15, 2022, and July 15, 2025, a total of 16 patients were enrolled (Figure 1A). The trial was discontinued owing to insufficient patient enrollment within the allocated time frame. The baseline characteristics of the enrolled patients are listed in Table 1. The median age was 51 years (range, 44–71). Most patients were male (62.5%), had metachronous metastasis (68.75%), an Eastern Cooperative Oncology Group (ECOG) performance score of 0 (75.0%), and multiple intrahepatic tumors (> 3, 93.75%). Half of the patients had previously received PD‐1 blockade therapy. All patients were negative for *BRAF* mutation.

**TABLE 1:** Patient baseline characteristics.

Category	Whole cohort *N*, (%)
Age, years, median (range)	51 (44–71)
Sex	
Male	10 (62.5)
Female	6 (37.5)
Tumor origin	
Cutaneous	6 (37.5)
Mucosal	6 (37.5)
Uveal	4 (25.0)
ECOG	
0	12 (75.0)
1	4 (25.0)
LDH level	
Normal	8 (50.0)
Elevated	8 (50.0)
Metastatic onset	
Synchronous	5 (31.25)
Metachronous	11 (68.75)
Number of intrahepatic metastasis	
1–3	1 (6.25)
> 3	15 (93.75)
Intrahepatic tumor size (cm)	
< 3	8 (50.0)
≥ 3	8 (50.0)
Number of metastatic sites	
1	3 (18.75)
2	5 (31.25)
3	3 (18.75)
> 3	5 (31.25)
Previous treatments for liver metastasis	
None	7 (43.75)
Chemotherapy	2 (12.50)
αPD1	2 (12.50)
αPD1+TKI	2 (12.50)
αPD1+chemotherapy	1 (6.25)
Others^a^	2 (12.50)
Previous PD‐1 blockade therapy	
Absent	8 (50.0)
Present	8 (50.0)

^a^ Others: αPD‐L1, B7‐H3‐targeting ADC.

### 2.2 ECOG, PD‐1, and Tyrosine Kinase Inhibitor (TKI)

During the 6‐month trial period, the median number of co‐ablation procedures was 2.5 (range, 1–6). Technical success was achieved in 68 of 69 targeted intrahepatic lesions (98.6%) after a single cryoablation session. Local tumor progression (LTP) at ablated lesions was observed in 0 of 68 (0%) technically successful ablations during the follow‐up period, indicating durable local control at the treated sites. The median number of sintilimab cycles was 3 (range, 1–7). For the three patients who completed the study treatment, sintilimab was continued until disease progression or loss of follow‐up. As of August 31, 2025, the median duration of follow‐up was 17.27 months.

### 2.3 Efficacy

**FIGURE 2:**
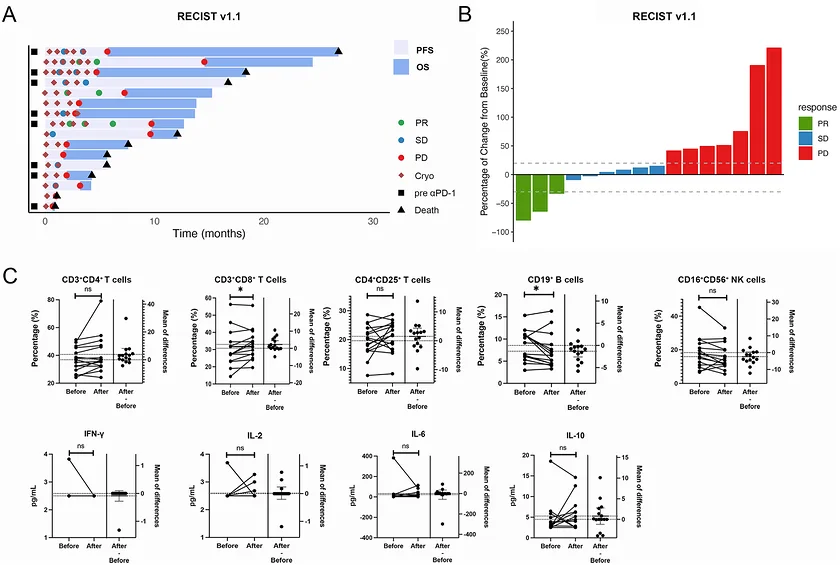
Treatment response and changes of immune markers. (A) Treatment exposure and response duration per RECIST v1.1. (B) Best percent change from baseline in target lesions per RECIST v1.1. (C) Changes of immune cell subsets and Th1‐ and Th2‐related cytokines in the peripheral blood before and 3 weeks after combination treatment. PD, progression disease; PR, partial response; SD, stable disease.

Of the 16 patients enrolled, three had a confirmed partial response according to Response Evaluation Criteria in Solid Tumors (RECIST) v1.1, with an ORR of 18.75% (95% confidence interval [CI]: 6.59–43.00). The overall disease control rate was 56.25%. Among patients with previous PD‐1 blockade therapy, one (1/8, 12.5%) had a response, and among those without previous PD‐1 blockade therapy, two (2/8, 25.0%) had a response. In patients who achieved a response, the median time to response was 2.26 months (range, 2.03–3.13), and the median duration of response was 9.73 months (range, 7.27–19.93) (Table 2 and Figure 2A,B).

**TABLE 2:** Tumor response (*n* = 16).

Variables	RECIST 1.1 (*n* = 16)
Best objective response	
Complete response	0 (0.0%)
Partial response	3 (18.75%)
Stable disease	6 (37.50%)
Progressive disease	7 (43.75%)
Objective response rate, *n* (%)	3 (18.75%)
Disease control rate, *n* (%)	9 (56.25%)
PFS, months, median (95% CI)	3.94 (2.77–14.30)
6‐month PFS rate	31.25%
12‐month PFS rate	18.75%
Liver‐specific PFS, months, median (95% CI)	5.05 (2.93–NR)
6‐Month liver‐specific PFS rate	37.50%
12‐Month liver‐specific PFS rate	31.25%
18‐Month liver‐specific PFS rate	11.71%

Abbreviations: CI, confidence interval; NR, not reached; PFS, progression‐free survival; RECIST, Response Evaluation Criteria in Solid Tumors.

**FIGURE 3:**
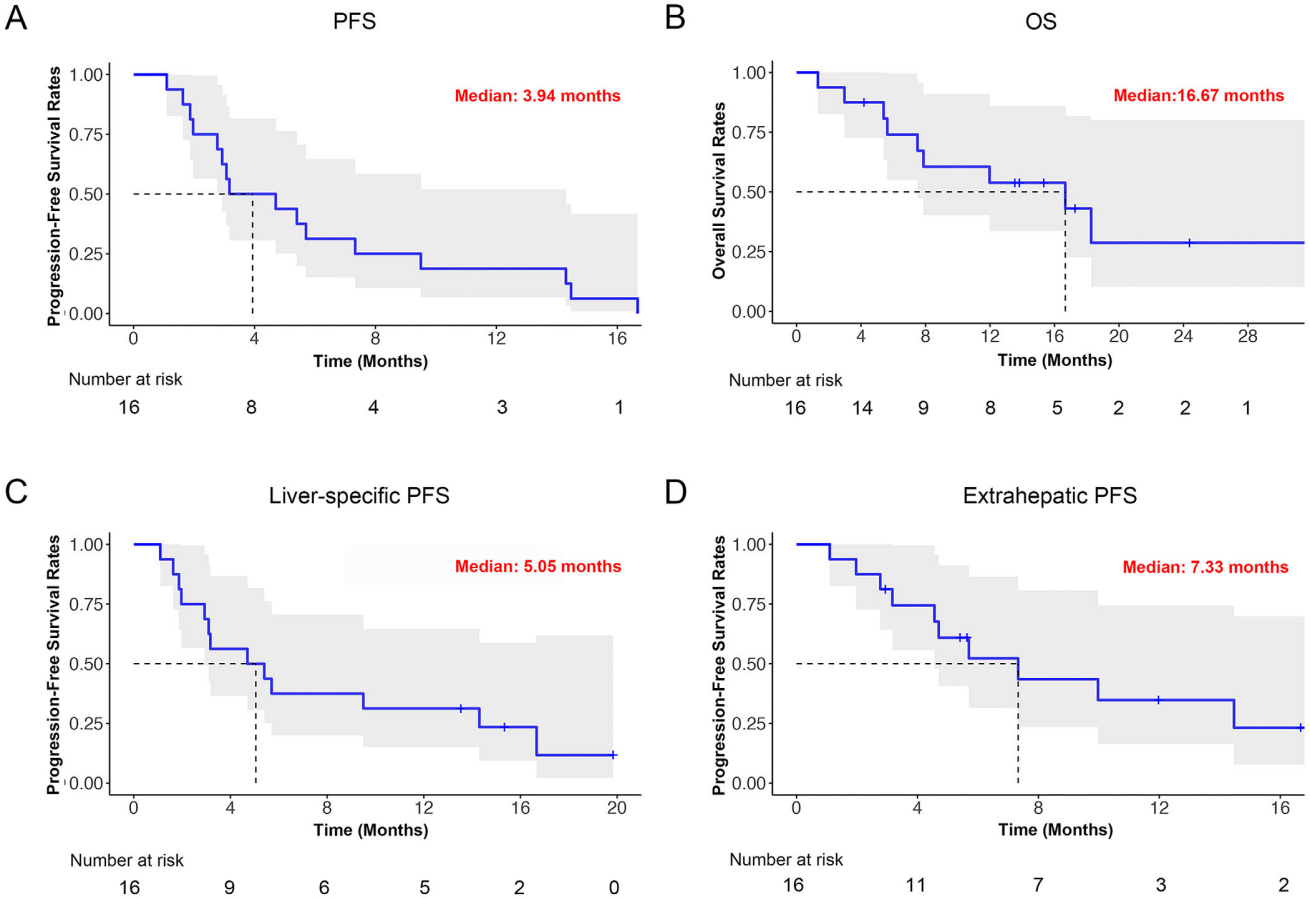
Survival analysis. (A) Kaplan–Meier survival curves of progression‐free survival (PFS) per RECIST v1.1. (B) Kaplan–Meier curves of overall survival. (C) Kaplan–Meier curves of liver‐specific PFS per RECIST v1.1. (D) Kaplan–Meier curves of extrahepatic PFS per RECIST 1.1.

**FIGURE 4:**
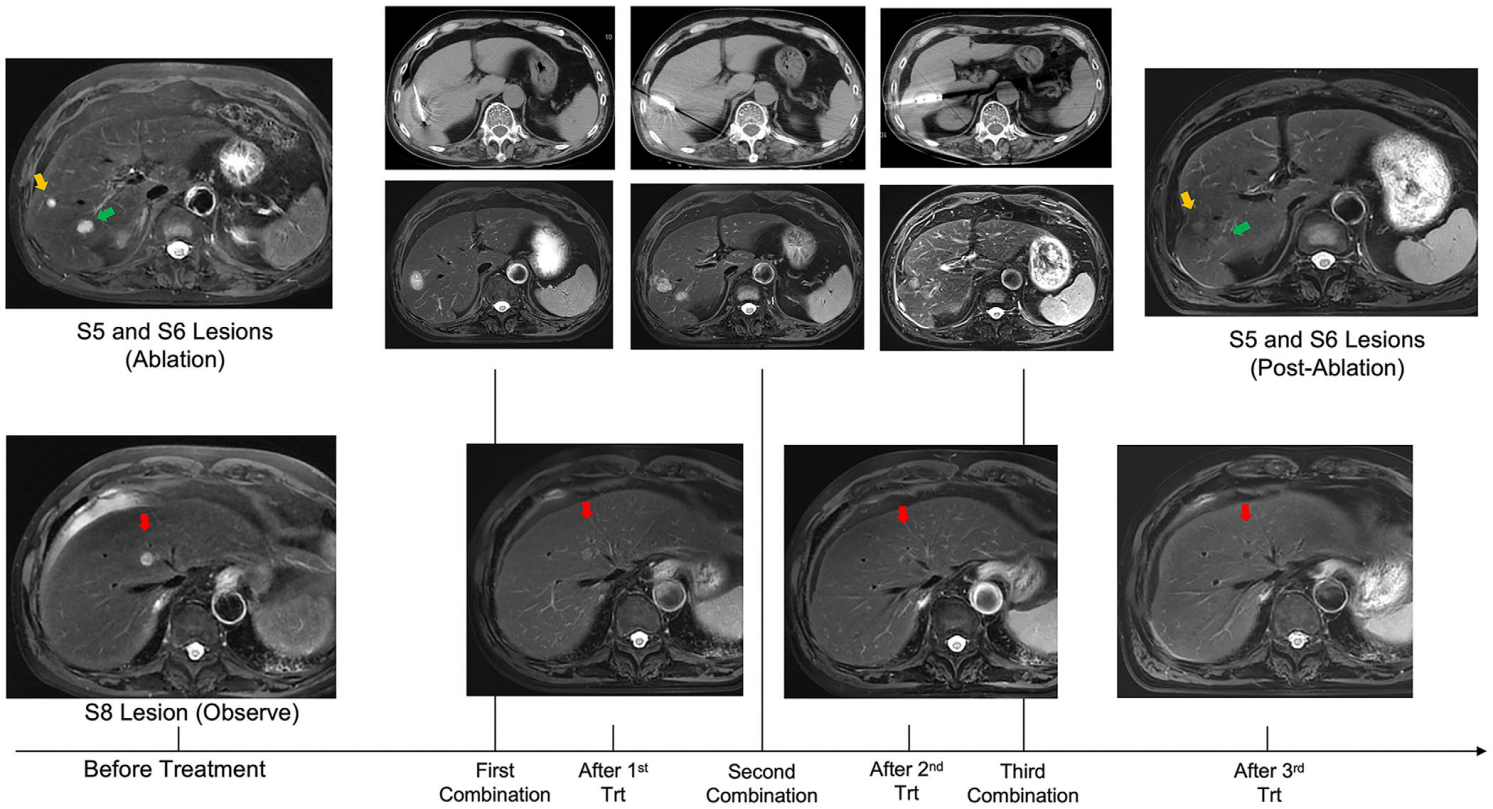
Representative case. An adult patient is diagnosed with anal melanoma (*BRAF* mutation negative) and has local lymph node metastasis in November 2023. After five cycles of albumin paclitaxel and carboplatin chemotherapy, the patient develops multiple liver metastasis. After receiving three cycles of combined co‐ablation and sintilimab treatment, the target liver metastases (unablated) shrink and show no activity.

The median PFS was 3.94 months (95% CI: 2.77–14.30), with 6‐ and 12‐month PFS rates of 31.25% and 18.75%, respectively (Figure 3A). The median liver‐specific PFS time was 5.05 (95% CI: 2.93–not reached [NR]) months, and the median extrahepatic PFS time was 7.33 (95% CI: 4.56–NR) months. The median overall survival (OS) was 16.67 months (95% CI: 7.50–NR), with 6‐, 12‐, and 24‐month OS rates of 74.0%, 53.8%, and 28.7%, respectively (Figures 3B–D and Figure 4).

### 2.4 Exploratory Immune Correlative Studies

Immune correlative test result data from peripheral blood samples collected before and after the first combination therapy were available from 15 of the 16 patients. The proportion of CD3^+^CD8^+^ T cells increased significantly (*p* = 0.042), whereas that of CD19^+^ B cells decreased (*p* = 0.046) 3 weeks after the combination therapy. The differences in regulatory T cells, CD3^+^CD4^+^ T cells, and NK cells (CD16^+^CD56^+^) were not significant; furthermore, no significant changes in IL‐2, IL‐6, IL‐10, and IFN‐γ levels were detected (Figure 2C). In an exploratory analysis, responders (*n* = 3) showed numerically greater increases in CD3^+^CD8^+^ T cells (mean change +5.07% vs. +1.57%) and greater decreases in CD19^+^ B cells (−2.25% vs. −1.09%) compared with non‐responders (*n* = 12), although these differences were not significant (Figure S1).

**FIGURE 5:**
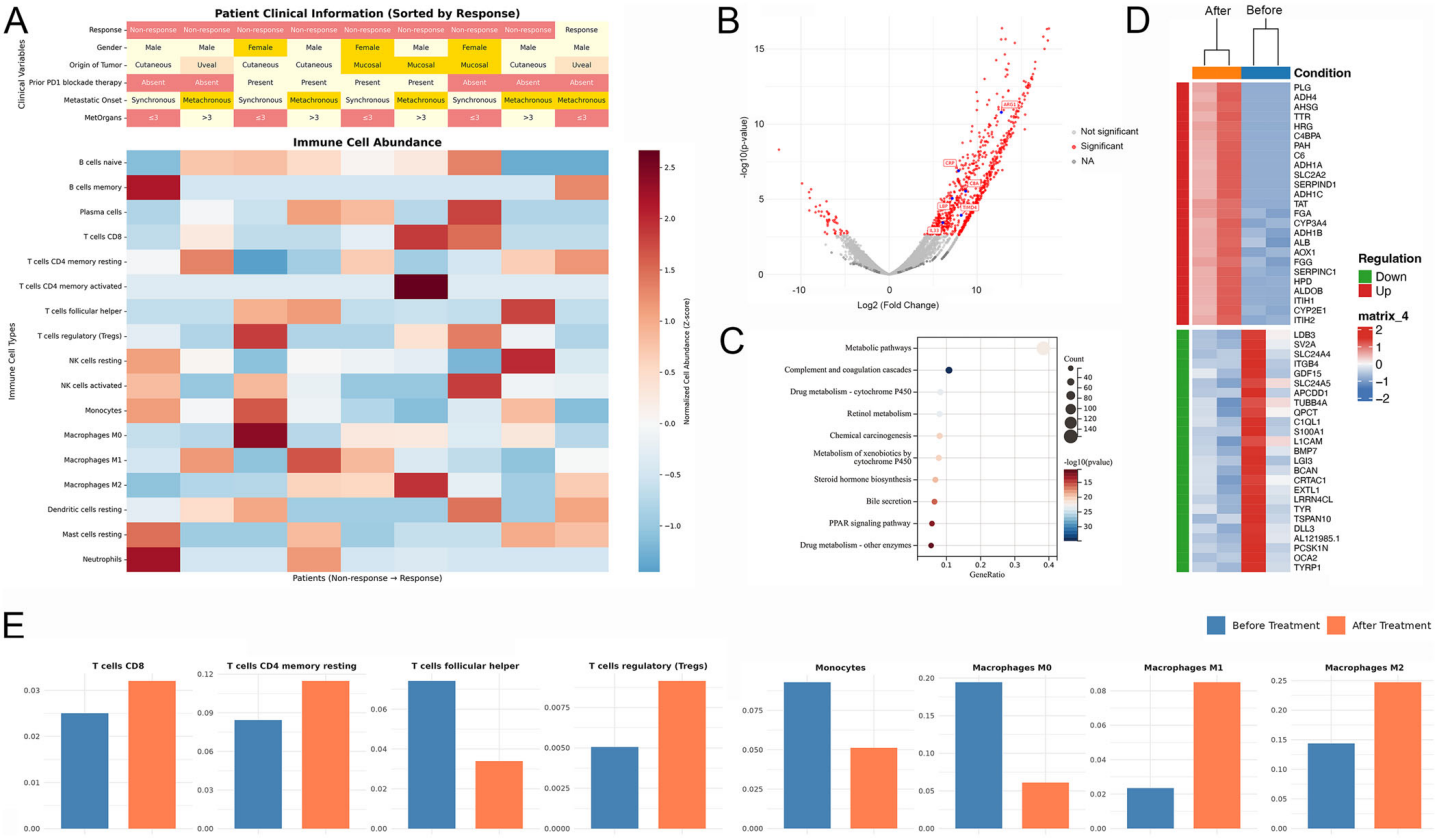
RNA seq of liver biopsies shows the key pathways activated after combined therapy. (A) The heatmap of patients (*n* = 9) with RNA seq data prior combined treatment. (B) After combined therapy, two patients undergo re‐biopsy and samples are used for RNA sequencing. A total of 833 differentially expressed genes (DEGs) are identified: 787 upregulated and 46 downregulated. (C) The top pathways in which the DEGs are involved based on Kyoto Encyclopedia of Genes and Genomes analysis include metabolic pathways, complement and coagulation cascades, and drug metabolism. (D) Genes with the largest fold changes between paired pre‐ and post‐treatment samples (*n* = 2; descriptive only, no statistical analysis applied). (E) The changes of key immune cell infiltrates after combined therapy based on CIBERSORTx. *n* = 2 per time point (paired); statistical comparison is not applicable.

Among the patients who received trial treatment, nine (56.3%) had pretreatment liver biopsy samples and two (12.5%) had paired biopsy samples after two cycles of treatment (Figure 5A). A total of 833 differentially expressed genes (DEGs) were identified based on analysis of paired samples, with 787 up‐regulated and 46 down‐regulated (Figure 5B). The top pathways in which these DEGs were involved based on KEGG Kyoto Encyclopedia of Genes and Genomes (analysis) included metabolic pathways, complement and coagulation cascades, and drug metabolism (Figure 5 C,D). In addition, trends toward an increase of CD8^+^ T cells, M1 macrophages, and M2 macrophages after treatment were observed; however, statistical significance was not reached due to the limitation in sample size (Figure 5E). With only two paired samples, these findings should be considered as strictly exploratory and hypothesis‐generating, requiring validation in future studies.

### 2.5 Post‑Progression Therapy

Subsequent treatments after disease progression are detailed in Table S1. In brief, 10 of 16 patients (62.5%) received further systemic and/or locoregional therapy, most commonly transarterial chemoembolization combined with anti‐PD‐1 antibodies and lenvatinib, while the remaining six (37.5%) received best supportive care only.

### 2.6 Safety

Procedure‐related complications were rare. According to the SIR classification, one patient (6.3%) experienced a grade B complication (abdominal bleeding managed conservatively with observation; hospitalization < 48 h). No major complications (grades C–F) or other minor complications were observed.

**FIGURE 6:**
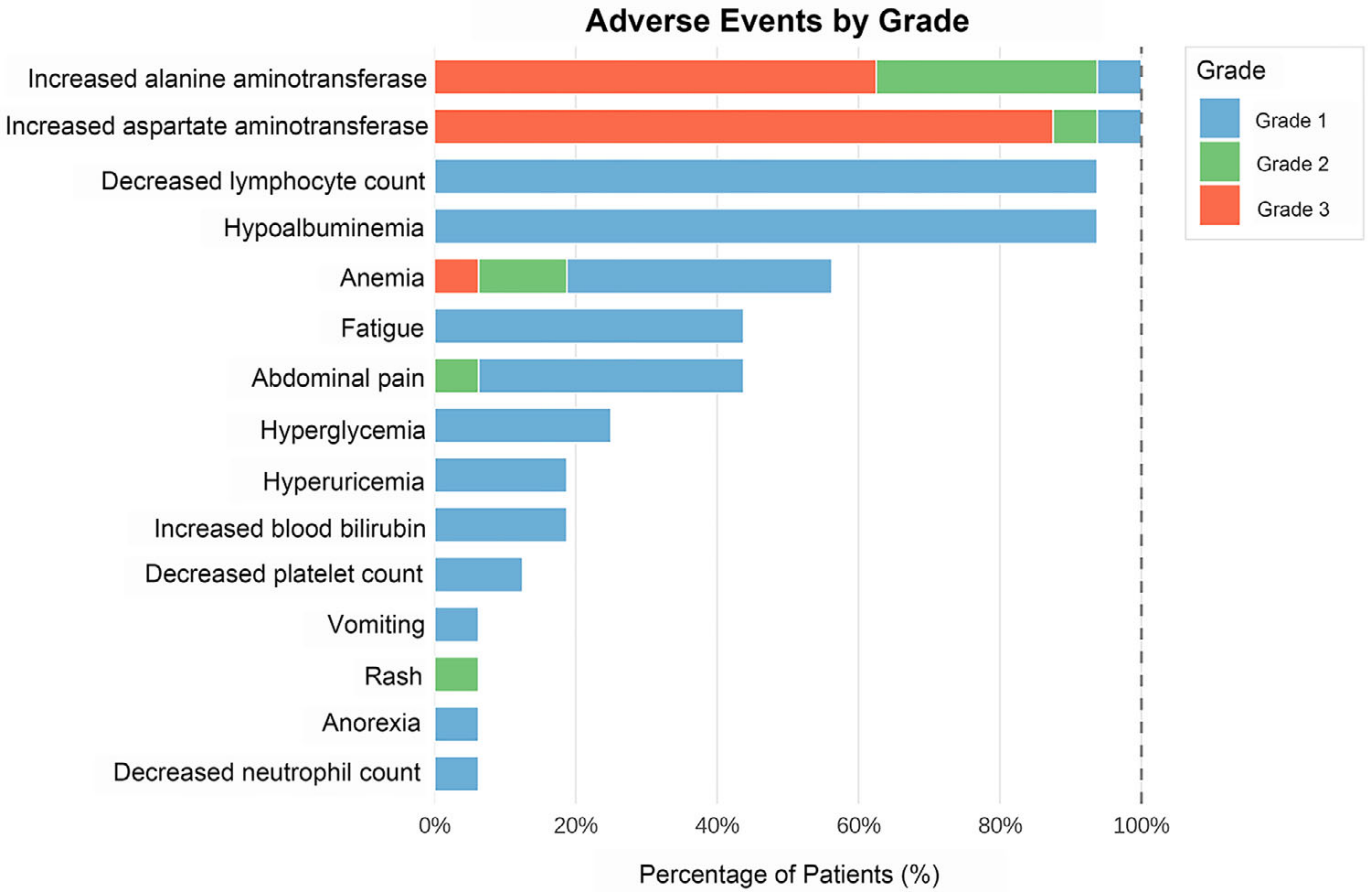
Graphical summary of treatment‐related adverse events.

The most frequent treatment‐related adverse events (AEs) were increased aspartate aminotransferase (AST) levels (16 [100.0%]), increased alanine aminotransferase (ALT) levels (16 [100.0%]), decreased lymphocyte count (16 [100.0%]), hypoalbuminemia (15 [93.75%]), anemia (16 [100.0%]), increased blood bilirubin levels (16 [100.0%]), decreased platelet count (16 [100.0%]), hyperuricemia (16 [100.0%]), abdominal pain (7 [43.75%]), hyperglycemia (4 [25.0%]), and fatigue (7 [43.75%]; Table S2; Figure 6). Treatment‐related AEs of grade 3 were observed in 14 (87.5%) patients, with the predominant events being increased AST levels (14 [87.5%]) and increased ALT levels (10 [62.5%]). No grade 4 or higher AEs occurred. Among the 14 patients with grade 3 AST elevation and 10 with grade 3 ALT elevation, all episodes peaked within 48 h after co‐ablation and resolved to less than grade 1 with standard hepatoprotective therapy (Table S3). No patient developed delayed transaminase elevation (>7 days after sintilimab), required corticosteroids, or discontinued treatment due to hepatotoxicity. Concurrent grade ≥ 3 hyperbilirubinemia or coagulopathy was not observed. No patients had dose reduction or discontinued treatment owing to AEs. No patients experienced serious irAEs.

## 3 Discussion

The CryoCheck‐001 trial was the first prospective study to assess the efficacy of co‐ablation combined with PD‐1 blockade therapy for the treatment of liver‐metastatic melanoma, which demonstrated the safety and efficacy of this combination treatment, with no serious irAEs identified.

Compared to conventional argon–helium cryoablation, co‐ablation rapidly cools the target lesion to −196°C, exhibiting stronger cooling capacity, higher freezing efficiency, and a larger single‐needle freezing zone [19]. Currently, co‐ablation technology has been applied for the treatment of various solid tumors, including lung cancer [20], melanoma [12], and liver cancer [19]. However, few studies have reported its potential in inducing antitumor immunity, also known as the abscopal effect. In the present study, the 6‑month PFS rate was 31.25% (95% CI: 15.1%–64.6%). Although this rate has not reached the 40.0% target, the trial was terminated early and enrolled only 16 of 45 planned patients, limiting the precision of the estimate and precluding formal confirmatory inference. Importantly, the observed rate exceeded the historical 20% benchmark for PD‑1 monotherapy [21], and the median PFS of 3.94 months was comparable to that reported in other cryoablation‐immune checkpoint inhibitor (ICI) combination studies in this population. These findings, while preliminary, suggest that co‐ablation may augment the antitumor activity of PD‑1 blockade and provide a rationale for future adequately powered trials. The observed ORR was 18.75%, which was higher than that shown in previous studies on PD‐1 blockade monotherapy for liver‐metastatic melanoma [22]. Notably, one patient with previous anti‐PD‐1 antibody tolerance also achieved a response. Mooradian et al. reported results from a phase II trial that evaluated the safety and efficacy of cryoablation and post‐progression immune checkpoint inhibition in 17 patients with metastatic melanoma, with an ORR of 23.5% and a disease control rate of 41%. Their study supported the use of cryoablation in the treatment of metastatic melanoma after ICI treatment failure [23]. Similarly, our study results support the use of co‐ablation in the treatment of liver‐metastatic melanoma, regardless of whether an ICI has been administered.

Nevertheless, a closer examination of progression patterns revealed an important discrepancy that median extrahepatic PFS (7.33 months) exceeded liver‐specific PFS (5.05 months). This discrepancy likely reflects the liver's inherently tolerogenic microenvironment, which may render intrahepatic metastases more resistant to immune control even after systemic T‐cell priming. Methodological factors may also contribute to this discrepancy because hepatic lesions were assessed using high‐resolution magnetic resonance imaging more frequently, potentially leading to earlier detection of progression compared with extrahepatic lesions. Importantly, this discordance highlights intrahepatic lesions as the major obstacle to efficacy. Accordingly, future treatment strategies should intensify local hepatic control, such as via extended or repeat ablation, or by adding hepatic arterial infusion or anti‐angiogenic therapy to counteract the immunosuppressive microenvironment.

The TBNK test of peripheral blood reflects the impact of the combination treatment on the systemic immune status in patients [24, 25], and dynamic monitoring is essential for optimal cancer management [26]. In this trial, a significantly increased proportion of CD3^+^CD8^+^ T cells was observed 3 weeks after the first combination treatment. Shewarega et al. conducted a preclinical study on the impact of incomplete cryoablation in HCC mouse models and observed an increase in CD8^+^ T cells in residual tumors [27]. Additionally, Chandra et al. observed a similar phenomenon in mice with metastatic breast cancer (4T1) [28]. Our study showed that co‐ablation, which integrates both cryo‐ and high‐temperature ablation technologies, may enhance anti‐tumor immunity in a manner similar to that of cryoablation alone. In our previous study on liver‐metastatic melanoma, CD19^+^ cells were identified as an unfavorable marker of treatment response to ICI‐based immunotherapy [7]. Li et al. found that STING‐induced regulatory B cells could compromise NK function in cancer immunity [29], while Lu et al. showed that complement signaling determined the roles of B cells in anti‐tumor immunity, highlighting the multifaceted mechanisms underlying B‐cell‐mediated immune response [30]. In this trial, a significant decrease in B‐cell abundance was observed in peripheral blood, suggesting that the combination treatment may enhance anti‐tumor immunity in a B‐cell‐dependent manner. However, because this finding was derived from the analysis of peripheral blood without paired tumor biopsies, it remains uncertain whether it reflects changes within the hepatic tumor microenvironment. Therefore, this B‐cell hypothesis requires validation in future studies with sequential tissue sampling.

Recent mechanistic studies provide a compelling framework for our findings. Gu et al. demonstrated that cryoablation enhances tumor immunogenicity and recruits CD8^+^ effector T cells, yet these cells initially remain trapped in the peritumoral stroma interacting with myofibroblastic CAFs; the addition of anti‐angiogenic therapy (lenvatinib) was required to normalize vessels and remodel the stroma, enabling T‐cell penetration into tumor nests [13]. Concurrently, the BOOSTER trial revealed that cryoablation may confer superior survival over thermal ablation, potentially via enhanced type I interferon (IFN‐ɑ) responses [14], aligning with our observed increase in CD3^+^CD8^+^ T cells and highlighting the immunostimulatory advantage of the co‐ablation system's freezing component. Collectively, these data suggest that co‑ablation effectively primes systemic T‑cell immunity.

Despite this immunological priming, the current ORR of the combination of co‐ablation and sintilimab in liver‐metastatic melanoma remains relatively low, being considerably lower than the 30%–40% response rate observed in patients without liver metastases, which indicates substantial room for future improvement. The CheckMate‐067 trial compared the efficacy of dual immunotherapy (nivolumab+ipilimumab), anti‐PD‐1 monotherapy (nivolumab), and anti‐CTLA‐4 monotherapy (ipilimumab) in patients with advanced melanoma [31]. With a 10‐year follow‐up, the study showed that the dual immunotherapy achieved significantly improved PFS and OS compared to either monotherapy [32, 33]. Consequently, dual immunotherapy has been recommended as a first‐line treatment option in the European and NCCN guidelines [34, 35]. The combination of co‐ablation with dual immunotherapy, including the innovative drugs iparomlimab and tuvonralimab [36, 37], represents a highly feasible treatment strategy.

Beyond dual immunotherapy, combining cytotoxic chemotherapy with anti‐angiogenic agents may offer another avenue to enhance efficacy [38]. Dacarbazine is a classic chemotherapeutic agent for advanced (metastatic or unresectable) melanoma and has long been considered the standard first‐line regimen. Intravenous dacarbazine chemotherapy has been reported to achieve a response rate of 26.3% in Asian patients with metastatic melanoma [39]. Lenvatinib is a multi‐targeted TKI [40]. The LEAP‐004 study demonstrated that the combination of lenvatinib and pembrolizumab achieved a response rate of 21.4%, with a median PFS of 4.2 months and median OS of 14 months, even after resistance to first‐line immunotherapy (anti‐PD‐1 or anti‐CTLA‐4 antibodies) [41]. Therefore, combining chemotherapy and anti‐angiogenic targeted agents with cryoablation and ICIs holds the potential for enhancing treatment efficacy in liver‐metastatic melanoma [42]. However, given the complexity and potential toxicity of such multi‐agent combinations, future trials should incorporate baseline CD8^+^ T‐cell infiltration or PD‐L1 expression for patient selection [43] and include sequential biopsies to validate mechanistic predictions. For anti‐angiogenic combinations, PFS with correlative immune endpoints is an appropriate primary outcome.

The 87.5% incidence of grade 3 transaminase elevation warrants careful interpretation. In all affected patients, the peak occurred within 48 h post‐ablation and rapidly improved with hepatoprotective therapy, without any case of delayed (> 7 days after sintilimab) elevation. This temporal pattern, together with the absence of concurrent grade 3 hyperbilirubinemia or coagulopathy, strongly suggests that the predominant mechanism involves acute hepatocellular injury secondary to the thermal‐mechanical effect of co‑ablation on peritumoral liver parenchyma, rather than immune‐mediated hepatitis. Immune‐related hepatitis typically presents with a delayed onset (≥ 7 days after PD‐1 blockade) and often requires corticosteroid intervention, neither of which was observed in our cohort. Although we cannot exclude a contributory role of sintilimab, the absence of classic features of immune‐related hepatitis and the prompt reversibility with supportive care indicate that the combination regimen does not increase the risk of severe immunotherapy‐related liver toxicity.

### 3.1 Limitations of the Study

This study had certain limitations. First, the lack of a control arm in our single‐arm design makes it difficult to definitively attribute the observed benefits solely to the addition of co‐ablation. Second, although encouraging results were observed, the sample size was limited, and the efficacy of co‐ablation and PD‐1 blockade therapy warrants further validation in larger prospective cohorts. Third, an in‐depth analysis of the tumor microenvironment after treatment was lacking owing to the scarcity of tumor samples. Fourth, although our protocol did not exclude patients with *BRAF* mutation to maximize generalizability, all enrolled patients were *BRAF* mutation negative. Thus, our results may not apply to patients with *BRAF* mutation failing targeted therapy. Future phase III trials should adopt a pragmatic design without mandatory *BRAF* testing but incorporate pre‐planned subgroup analyses to evaluate potential differential effects. Finally, the early termination with only 16 of 45 enrolled patients substantially reduced statistical power and increased the risk of Type II error. The observed PFS estimates should therefore be interpreted with caution because small sample sizes may yield unstable effect sizes. Nonetheless, the consistent results (exceeding the historical benchmark and aligning with other ablation‐ICI studies) support the biological plausibility of our findings. These results are hypothesis‐generating and warrant validation in larger, confirmatory trials. Despite these limitations, our findings provide a rationale for future larger scale trials that incorporate comprehensive biomarker analyses to validate and build upon these results.

## 4 Conclusion

Co‐ablation plus sintilimab demonstrates a manageable safety profile and suggests preliminary antitumor activity in patients with liver‐metastatic melanoma, warranting further validation in larger, confirmatory trials.

## 5 Materials and Methods

### 5.1 Study Design and Patients

The CryoCheck‐001 study was a single‐arm clinical trial. The inclusion criteria were as follows: (1) pathologically confirmed metastatic malignant melanoma after failure of standard therapy; (2) presence of liver metastases, with at least one lesion suitable for cryoablation after evaluation; (3) largest diameter of intrahepatic metastases < 5 cm; (4) ECOG performance status score of 0 or 1; (5) no history of autoimmune disease; and (6) life expectancy greater than 3 months. The key exclusion criteria were as follows: (1) prior local therapy for liver metastases; (2) Child–Pugh score ≥ 7; (3) evidence of liver function decompensation; (4) clinically unstable central nervous system metastasis; and (5) previous irAEs of grade 3 or above after ICI treatment (Figure 1A). All patients provided written informed consent. The study protocol was approved by the Institutional Review Board of Sun Yat‐sen University Cancer Center (Guangzhou, China‐based institution; B2022‐261‐02) and is registered with ChiCTR.org.cn, ChiCTR2200061591. This study was reported in accordance with the Strengthening the Reporting of Observational Studies in Epidemiology (STROBE) guidelines.

### 5.2 Combination Treatment Plan

After enrollment, the combination treatment protocol was as follows. During the first treatment cycle, co‐ablation was performed first, followed by intravenous sintilimab (200 mg) within 3 days after the ablation procedure. This sequence (ablation before PD‐1 blockade) was consistently applied across all enrolled patients. Subsequently, sintilimab was administered every 3 weeks, and the decision to perform co‐ablation in the second and subsequent cycles was made by a multidisciplinary team based on dynamic evaluation of intrahepatic lesions and the status of each patient. A maximum of eight cycles of combination treatment were scheduled within the first 6 months. If the disease did not progress, patients completed up to 6 months of combination therapy (maximum of eight cycles). Completion of the 6‐month treatment period marked the end of the trial treatment (Figure 1B). Subsequent treatment plans were determined through shared decision‐making between the physician and the patient.

### 5.3 Co‐Ablation

Co‐ablation was performed using the Hygea Co‐ablation System (Hygea Medical Technology Co. Ltd, Beijing, China), which is equipped with two types of probes (model 12G, length 150 or 180 mm). For each procedure, one to three intrahepatic lesions with the largest diameter < 5 cm were ablated. Target lesions were selected and treated with full ablative intent based on critical technical factors (e.g., proximity to large vessels and ease of access). The procedures were performed by experienced interventional radiologists (W.F. and L.S., with 18 and 10 years of experience, respectively). In the absence of intrahepatic progression, co‐ablation was continued until all intrahepatic lesions > 5 mm in diameter that could be safely accessed were ablated. Typically, one probe was used for lesions with a diameter < 3 cm, and one to two probes were used for lesions with a diameter of 3–5 cm. To accurately evaluate the effect of combination therapy on tumors, patients with extrahepatic metastases that could serve as target lesions for efficacy monitoring underwent co‐ablation of all intrahepatic lesions. For patients with only intrahepatic metastases, at least one intrahepatic lesion was left unablated and served as a target lesion for observation.

Under CT guidance (Brilliance CT Big Bore; PHILIPS), probes were positioned within the target tumor after administration of local anesthesia with 2% lidocaine. Each ablation comprised two 8–15 min freeze cycles, interspersed with a 2‐min passive thaw and a 3‐min active heating phase. Overlapping ablations using the same parameters were conducted when necessary. Technical success was defined as ice‐ball extension ≥ 5 mm beyond the tumor margin. Vital signs were monitored continuously during the procedure, and a non‐contrast CT scan was obtained immediately after probe removal to screen for early complications. Routine blood tests and biochemical tests were obtained within 24 h after the treatment.

### 5.4 PD‐1 Blockade Therapy

For the administration of sintilimab, a homogeneously mixed solution of 200 mg sintilimab and 100 mL normal saline was injected slowly over 30–60 min.

### 5.5 Follow‐Up and Endpoint Assessment

Patients were followed at clinic visits every 3 weeks during the study treatment. Tumor response assessments were conducted according to RECIST v1.1 [44]. Response evaluation was performed on pre‑specified target lesions that were not subjected to co‑ablation. For patients with both intrahepatic and extrahepatic metastases, extrahepatic target lesions were preferentially selected whenever feasible. For patients with intrahepatic lesions only, at least one measurable intrahepatic lesion was deliberately spared from ablation to serve as a target lesion for response monitoring. All target lesions were selected at baseline, and serial measurements were performed using consistent imaging modalities throughout follow‑up. The first efficacy evaluation was performed at (6 ± 1) weeks after treatment initiation, with subsequent assessments every 6 weeks. The response assessment was completed by two experienced radiologists (L.S. and L.L., with 10 and 7 years of experience, respectively).

The primary endpoint was the 6‐month PFS rate, defined as the proportion of patients without disease progression at 6 months after enrollment according to RECIST v1.1. Secondary endpoints included ORR per RECIST v1.1, OS, PFS, and safety. PFS was defined as the time from initiation of study treatment to disease progression or death from any cause, and OS was defined as the time from initiation of study treatment to death from any cause. LTP was defined as new or progressive nodular enhancement at the ablation margin or within the ablation zone on follow‐up contrast‐enhanced imaging. Procedure‐related complications were classified according to the Society of Interventional Radiology (SIR) classification system (minor: A–B; major: C–F). AEs were graded according to the National Cancer Institute Common Terminology Criteria for Adverse Events version 4.0.

Exploratory endpoints included liver‐specific PFS, extrahepatic PFS, and changes in the immune correlative test of peripheral blood after combination treatment. Liver‐specific PFS was defined as the time from initiation of study treatment to progression of liver metastases or death from any cause. The extrahepatic PFS was defined as the time from initiation of study treatment to progression of extrahepatic lesions.

### 5.6 Immune Correlative Studies

Blood samples for immune correlative analysis were available from 15 patients (93.75%). The frequencies of T cells (CD3^+^), cytotoxic T cells (CD3^+^CD8^+^), regulatory T cells (CD4^+^CD25^+^), B cells (CD19^+^), and NK cells (CD3^−^CD16^+^CD56^+^) were measured using multicolor flow cytometry (BD multitest 6‐color TBNK Reagent plus BD CD25 Assay Kit). Fresh blood samples were centrifuged over a Ficoll‐Paque PLUS (Cytiva) density gradient to isolate PBMCs. For immunophenotyping, approximately 1 × 10^6^ PBMCs were stained with a panel of fluorescently conjugated antibodies at pre‐optimized concentrations for 30 min at 4°C in the dark. The antibody panel included anti‐CD3 (FITC, clone SK7), anti‐CD4 (^21^PE‐Cy7, clone SK3), anti‐CD8 (APC‐Cy7, clone SK1), anti‐CD19 (^22‐24^APC, clone SJ25C1), anti‐CD16 (^13‐16^PE, clone B73.1), anti‐CD56 (^13‐16^PE, clone NCAM16.2), and anti‐CD25 (PE, clone 2A3). The stained cells were washed twice with PBS containing 1% BSA and then re‐suspended in fixation buffer. Data acquisition was performed on a flow cytometer (BD FACSCalibur), and a minimum of 50,000 events per sample were collected. Subsequent analysis was conducted using BD CellQuest Pro. Lymphocyte populations were first gated based on forward and side scatter properties. Levels of Th1 and Th2 cytokines, including interleukin (IL)‐2, IL‐4, interferon gamma (IFN‐γ), IL‐6, and IL‐10, were assessed using immunofluorescence analysis (Wellgrow Human Six Cytokine Assay Kit), with a detection limit of 2.5–5000 pg/mL.

### 5.7 Liver Biopsy and Bulk RNA Sequencing

The mRNA was captured using magnetic beads and then fragmented to generate cDNA. Sequencing libraries were constructed with the NEBNext Ultra RNA Library Prep Kit for Illumina (NEB, USA) per the manufacturer's protocol, with barcodes added during the process. Later, we used cBot Cluster Generation System using TruSeq PE Cluster Kit v3‐cBot‐HS (Illumina) to perform the clustering of the index‐coded samples. After cluster generation, the resulting libraries were sequenced on an Illumina system to generate 125 or 150 bp paired‐end reads. Differential expression analysis was performed using the edgeR R package. The *p* values were adjusted using the Benjamini and Hochberg method. An absolute fold change of 1.2 and *p*‐value of 0.05 were set as the threshold for identification of differentially expressed genes.

### 5.8 Immune Cell Infiltration

Immune cell infiltration was estimated from bulk RNA‑seq data using CIBERSORTx (https://cibersortx.stanford.edu/), a machine‑learning algorithm for transcriptome deconvolution [45]. The analysis was performed in B‑mode with batch correction enabled, using the LM22 signature matrix, which defines 22 human immune cell subtypes. Relative fractions of each immune cell type were calculated, and only samples with a *p*‐value < 0.05 were retained for downstream analysis.

### 5.9 KEGG Analysis

For gene set functional enrichment analysis, the latest KEGG pathway gene annotation (https://www.kegg.jp/kegg/rest/keggapi.html) was obtained as background. The R software package clusterProfiler (version 3.14.3) was utilized for enrichment analysis. A *p* value of < 0.05 and an FDR of < 0.25 were considered statistically significant.

### 5.10 Quantification and Statistical Analysis

This single‐arm, phase II trial was designed to evaluate the efficacy of cryoablation in combination with PD‐1 antibody therapy in patients with metastatic melanoma. The historical 6‐month PFS rate for PD‐1 antibody monotherapy, derived from the KEYNOTE‐151 trial, is 20.4% [21]. We estimated that the combination of cryoablation and PD‐1 therapy would increase the 6‐month PFS rate to 40.0%. We assumed that the time to progression follows a Weibull distribution, with the shape parameter set to 1.0. Using a two‐sided, log‐rank test with a significance level (*α*) of 0.05 and a power (1 − *β*) of 90%, a total of 45 patients was required to confirm that the 6‐month PFS rate of the combination therapy exceeds the historical benchmark of 20.4%, with a target performance of 40.0%. The trial was discontinued because of failure to enroll a sufficient number of patients within the allocated time frame.

All the enrolled patients were included for both efficacy and safety analyses. ORR and post hoc disease control rate are expressed as percentages, with 95% CIs calculated using the Clopper–Pearson method. PFS, OS, and post hoc liver‐specific PFS, extrahepatic PFS were estimated using the Kaplan–Meier method, and their 95% CIs were calculated using the Brookmeyer–Crowley method. All statistical analyses were performed using R 4.2.2 (The R Foundation for Statistical Computing).

## Author Contributions

Conception and design: L.S., L.L., S.C., and C.L. Data analysis and interpretation: L.S., L.L., and S.C. Resources: L.S. and W.F. Funding acquisition: W.F. Writing – original draft: L.S., L.L., S.C., C.L., R.T., L.X., and W.F. Writing – review and editing: All authors. Final approval of manuscript: All authors. L.S., Y.W., Y.L., H.Z., D.L,, and X.Z. have accessed and verified the data. W.J.F. is responsible for the decision to submit the manuscript. All authors have read and approved the final manuscript.

## Funding

This study was supported by the National Key R&D Program of China (2023YFC2414000).

## Ethics Statement

All patients provided written informed consent. The study protocol was approved by the Institutional Review Board of Sun Yat – sen University Cancer Center (Guangzhou, China based institution; B2022 – 261 – 02) and is registered with ChiCTR.org.cn, ChiCTR2200061591.

## Conflicts of Interest

The authors declare no conflicts of interest.

## Supporting information



Supporting FIle: 1 mco270997‐sup‐0001‐SuppMat.docx

## Data Availability

The authenticity of these data has been validated by uploading the critical raw data onto the Research Data Deposit public platform (www.researchdata.org.cn). The de‐identified data supporting the findings of this study are available from the corresponding author upon reasonable request, subject to institutional data‐sharing policies, and patient confidentiality requirements.
