# Supplementary material for: Efficacy and Safety of Co‐Ablation Combined With Aintilimab for Metastatic Melanoma to the Liver (CryoCheck‐001): A Phase II Trial
Source: MedComm (2020). 2026 Sep 15;7(10):e70997. doi: 10.1002/mco2.70997 (PMC13575768; doi:10.1002/mco2.70997)
Supplement: Supplementary file 1 [file MCO2-7-e70997-s001.docx]

***Original Article***

**Efficacy and safety of co-ablation combined with sintilimab for liver metastatic melanoma (CryoCheck-001): a phase II trial**

Lujun Shen, M.D.^1,2#^, Letao Lin, M.D.^1,2#^, Shuanggang Chen M.D.^1,2#^, Chen Li, M.S.^1,2#^, Ruizhi Tang, M.S.^1,2^, Lin Xie, M.D.^1,2^, Yujia Wang, M.D.^1,2^, Yunpeng Li, M.D.^1,2^, Hongliang Zou M.D.^3^, Dandan Li M.D.^2,4^*, Xiaoshi Zhang, M.D.^2,4^*, Weijun Fan, M.D.^1,2^*

^1^Department of Minimally Invasive Interventional Therapy, Sun Yat-sen University Cancer Center, Guangzhou 510060, China

^2^State Key Laboratory of Oncology in South China, Collaborative Innovation Center of Cancer Medicine, Sun Yat-sen University, Guangzhou 510060, China

^3^Department of Interventional Oncology, The First Affiliated Hospital of Sun Yat-Sen University, Guangzhou 510080, China

^4^Department of Biological Therapy Center, Sun Yat-sen University Cancer Center, Guangzhou 510060, China

**Figure S1.** The correlation between treatment response and changes of CD3^+^CD8^+^ T cells and CD19+ B cells in the CryoCheck-001 study.

**Table S1. Post-progression treatments received by patients after failure of co-ablation plus sintilimab**

| Patient ID | Best treatment response (RECIST 1.1) | PFS on trial (months) | Post-progression therapy (subsequent lines) |
| --- | --- | --- | --- |
| 1 | SD | 4.7 | DTIC + Toripalimab + Lenvatinib |
| 2 | SD | 5.7 | DTIC + Sintilimab +Lenvatinib |
| 3 | SD | 5.4 | BSC |
| 4 | PR | 14.47 | TACE + Sintilimab +Lenvatinib |
| 5 | PD | 1.63 | TACE + Pembrolizumab +Lenvatinib + Temozolomide |
| 6 | SD | 16.67 | BSC |
| 7 | SD | 9.5 | Pembrolizumab + Temozolomide |
| 8 | PR | 7.33 | Toripalimab + Bevacizumab + Albumin-bound paclitaxel |
| 9 | PD | 1.97 | TACE + Toripalimab + DTIC + Lenvatinib |
| 10 | SD | 3.17 | BSC |
| 11 | PD | 2.93 | BSC |
| 12 | PD | 3.07 | TACE + Toripalimab + Lenvatinib + Temozolomide |
| 13 | SD | 2.77 | TACE |
| 14 | PD | 1.87 | TACE+ Toripalimab + Temozolomide + Lenvatinib |
| 15 | PR | 14.3 | Tunlametinib |
| 16 | PD | 1.1 | BSC |

BSC, Best Supportive Care.

**Table S2. Treatment-related adverse effects of all grades**

| Categories | Any Grade | Grade 1 | Grade 2 | Grade 3 |
| --- | --- | --- | --- | --- |
| Increased aspartate aminotransferase | 16 (100.00%) | 1 (6.25%) | 1 (6.25%) | 14 (87.50%) |
| Increased alanine aminotransferase | 16 (100.00%) | 1 (6.25%) | 5 (31.25%) | 10 (62.50%) |
| Hypoalbuminemia | 15 (93.75%) | 15 (93.75%) | 0 (0.00%) | 0 (0.00%) |
| Decreased neutrophil count | 1 (6.25%) | 1 (6.25%) | 0 (0.00%) | 0 (0.00%) |
| Decreased lymphocyte count | 15 (93.75%) | 15 (93.75%) | 0 (0.00%) | 0 (0.00%) |
| Anemia | 9 (56.25%) | 6 (37.50%) | 2 (12.50%) | 1 (6.25%) |
| Decreased platelet count | 2 (12.50%) | 2 (12.50%) | 0 (0.00%) | 0 (0.00%) |
| Increased blood bilirubin | 3 (18.75%) | 3 (18.75%) | 0 (0.00%) | 0 (0.00%) |
| Proteinuria | 0 (0.00%) | 0 (0.00%) | 0 (0.00%) | 0 (0.00%) |
| Abdominal pain | 7 (43.75%) | 6 (37.50%) | 1 (6.25%) | 0 (0.00%) |
| Decreased white blood cell | 0 (0.00%) | 0 (0.00%) | 0 (0.00%) | 0 (0.00%) |
| Anorexia | 1 (6.25%) | 1 (6.25%) | 0 (0.00%) | 0 (0.00%) |
| Hyperglycemia | 4 (25.00%) | 4 (25.00%) | 0 (0.00%) | 0 (0.00%) |
| Hyperuricemia | 3 (18.75%) | 3 (18.75%) | 0 (0.00%) | 0 (0.00%) |
| Hypokalemia | 0 (0.00%) | 0 (0.00%) | 0 (0.00%) | 0 (0.00%) |
| Rash | 1 (6.25%) | 0 (0.00%) | 1 (6.25%) | 0 (0.00%) |
| Hypertension | 0 (0.00%) | 0 (0.00%) | 0 (0.00%) | 0 (0.00%) |
| Hand-foot syndrome | 0 (0.00%) | 0 (0.00%) | 0 (0.00%) | 0 (0.00%) |
| RCCEP | 0 (0.00%) | 0 (0.00%) | 0 (0.00%) | 0 (0.00%) |
| Diarrhea | 0 (0.00%) | 0 (0.00%) | 0 (0.00%) | 0 (0.00%) |
| Vomiting | 1 (6.25%) | 1 (6.25%) | 0 (0.00%) | 0 (0.00%) |
| Fatigue | 7 (43.75%) | 7 (43.75%) | 0 (0.00%) | 0 (0.00%) |
| Hematuria | 0 (0.00%) | 0 (0.00%) | 0 (0.00%) | 0 (0.00%) |
| Upper respiratory infection | 0 (0.00%) | 0 (0.00%) | 0 (0.00%) | 0 (0.00%) |
| Gingival hemorrhage | 0 (0.00%) | 0 (0.00%) | 0 (0.00%) | 0 (0.00%) |
| Fever | 0 (0.00%) | 0 (0.00%) | 0 (0.00%) | 0 (0.00%) |
| Ascites | 0 (0.00%) | 0 (0.00%) | 0 (0.00%) | 0 (0.00%) |
| Epistaxis | 0 (0.00%) | 0 (0.00%) | 0 (0.00%) | 0 (0.00%) |
| Cough | 0 (0.00%) | 0 (0.00%) | 0 (0.00%) | 0 (0.00%) |
| Headache | 0 (0.00%) | 0 (0.00%) | 0 (0.00%) | 0 (0.00%) |

**Table S3. Time to peak and resolution of Grade 3 liver enzyme elevations**

|  | Total number | Peak Within 1 week | Remission Within 1 week | Exceed 1 week |
| --- | --- | --- | --- | --- |
| Grade 3 AST | 14 | 14 | 14 | 0 |
| Grade 3 ALT | 10 | 10 | 10 | 0 |
